# Differential Infiltration of Immune Cells Driven by Tumor Heterogeneity Reveals Two Immune Subtypes in Lung Adenocarcinoma

**DOI:** 10.3389/fgene.2022.924781

**Published:** 2022-07-04

**Authors:** Liqiang Wang, Ying Song, Jing Bai, Wenjing Sun, Jingcui Yu, Mengdi Cai, Songbin Fu

**Affiliations:** ^1^ Key Laboratory of Preservation of Human Genetic Resources and Disease Control in China (Harbin Medical University), Ministry of Education, Harbin, China; ^2^ Laboratory of Medical Genetics, Harbin Medical University, Harbin, China; ^3^ Scientific Research Centre, The Second Affiliated Hospital of Harbin Medical University, Harbin, China

**Keywords:** lung adenocarcinoma, tumoral heterogeneity, immune, genomic, clinical prognosis

## Abstract

Intra-tumoral heterogeneity (ITH) is a critical factor leading to aggressive progression and response to immunotherapy in lung adenocarcinoma (LUAD). However, the relationship between ITH and immune cells in the tumor microenvironment (TME) has not been systematically elucidated. In the present study, we evaluated the ITH status of LUAD samples based on the mutational data obtained from The Cancer Genome Atlas database. First, we identified five key immune pathways with a significantly continuous downtrend among normal, low-heterogeneous, and high-heterogeneous samples and further excavated nine key immune cells related to the key immune pathways and tumor heterogeneity. Then, two immune subtypes were defined by a consensus clustering algorithm based on the infiltration of these immune cells. Differences between these two immune subtypes were remarkable, including alterations of tumor mutation burden and DNA copy number variation at the genomic level, various metabolic pathways, and the different clinical outcome, which was also validated in two independent Gene Expression Omnibus datasets. The results revealed that ITH was significantly associated with prognosis and infiltrating immune cells in the TME. Our study provides novel insights in understanding the relationship between ITH and immune cells and contributes to the immunotherapy of LUAD patients.

## Introduction

Lung adenocarcinoma (LUAD) is the most common histological subtype of lung cancer and the leading cause of cancer-related deaths worldwide (Bray et al., 2018). With the advances in diagnostic and therapeutic strategies, the prognosis of LUAD patients has been significantly improved. Recent studies have shown that LUAD emerges and develops under strong evolutionary pressures from immune, metabolic, and therapeutic factors (Zhang et al., 2019; Liu et al., 2020; Hu et al., 2021). Of note, this pressure promotes the diversification of malignant cells in the tumor microenvironment (TME), ultimately leading to intra-tumoral heterogeneity (ITH). Substantial evidence showed that ITH has a significant impact on the efficacy of various immunotherapies (Vitale et al., 2021). The infiltrating immune cells in the TME are critical factors affecting tumor growth and progression, thus being regarded as a promising target in anti-cancer immunotherapy (Shen and Ren, 2018; Feng et al., 2020). Therefore, characterization of the dynamic changes of infiltrating immune cells and ITH in the TME is of great importance for understanding immune response and provides novel insights into cancer immunotherapy.

In recent years, significant progresses have been made in understanding the formation and function of ITH. ITH is currently known to be crucial for cancer cell progression and is highly associated with abnormal genetic events including tumor mutational burden (TMB), aneuploidy, and metabolic dysfunction. Considerable genetic variations may occur in different regions within the same tumor, which is known as spatial ITH. Meanwhile, ITH within the same region of the tumor tissue may vary significantly over time with cancer progression. These indicated that ITH within the tumor may evolve in a spatial and temporal manner. Recent studies demonstrated that ITH might drive tumor development, which is an evolutionary process involving the interplay between tumor cells and the local immune microenvironment (Berglund et al., 2021). A report has shown that immune-related pathways can play a crucial role in the communication between tumor cells and immune cells in the TME (Hasso-Agopsowicz et al., 2018; Andresen et al., 2019). Thus, the plasticity of immune cells and major components in the TME may have been deeply influenced by ITH. Therefore, further studies about the impact of ITH on immune cells in the TME are essential for the development of effective immunotherapy.

In this study, we characterized distinct immune subtypes of LUAD by identifying ITH-related immune pathways and immune cells. We found that these immune subtypes were different in mutation burden, copy number variation (CNV), immune cell infiltration, significant mutation genes, and prognosis. Taken together, this study will provide theoretical evidence for personalized immunotherapy to improve the clinical outcome of LUAD patients.

## Materials and Methods

### Lung Adenocarcinoma Patient Cohorts

The transcriptome profile, somatic mutation data, CNV data, and clinical data of lung adenocarcinoma were all downloaded from The Cancer Genome Atlas (TCGA) database (http://cancergenome.nih.gov/) (Cancer Genome Atlas Research Network, 2008). The transcriptome profile included 59 normal samples and 535 cancer samples. Somatic mutation data covered 568 cancer samples. In addition, two other cohorts were obtained from the Gene Expression Omnibus (GEO) database (https://www.ncbi.nlm.nih.gov/geo/), including the GSE26939 dataset which has 116 patients and the GSE68465 dataset which has 433 patients.

### Calculation of Intratumoral Heterogeneity

The heterogeneous level of each tumor sample was evaluated by the MATH (Mutant-Allele Tumor Heterogeneity) Score (Mroz and Rocco, 2013) using the “infer Heterogeneity” method in the maftools package (Mayakonda et al., 2018) based on the mutational data of LUAD patients obtained from the TCGA database.

### Evaluation of the Activity Score for Each Immune Pathway

We collected the immune pathways and the pathway-related genes from the KEGG (https://www.genome.jp/kegg) database. In total, we obtained 20 types of immune pathways (Kanehisa et al., 2017). For each immune pathway, we evaluated its activity score in a sample group or in a sample by two approaches, as described below, based on the expression profile.

#### Evaluation of Immune Pathway Activity Score by the Jason W. Locasale Method

The activity scores of 20 immune pathways in each group (normal group, low-heterogeneous group, and high-heterogeneous group) were calculated with the method of Jason W. Locasale (Xiao et al., 2019). First, we transformed the FPKM normalization data to the TPM values, deleted those genes with more than 50% missing values across samples, and log2-transformed the expression profile. Then, for each immune pathway in a group, the activity score was calculated by the weighted summation of the relative expression value of genes in the pathway.

First, for each gene in immune pathways, we calculated its average expression level in the *j*-th heterogeneous group:

$$E_{i,j}=\frac{\sum_{k=1}^{n_{j}}g_{i,k}}{n_{j}},i\in 1\cdots M,j\in 1\cdots N$$

where $n_{j}$ is the number of samples in the *j*th heterogeneous group, $g_{i,k}$ is the expression level of the *i*-th gene in the *k*-th sample in this heterogeneous group, *M* is the number of immune-related genes, and *N* is the number of heterogeneous groups. In our study, *M* and *N* are 20 and 3, respectively.

Second, the relative expression level of the *i*-th gene in the *j*-th heterogeneous group was then defined as the ratio of $E_{i,j}$ to its average value over all heterogeneous groups:

$$r_{i,j}=\frac{E_{i,j}}{\frac{1}{N}\sum_{1}^{N}E_{i,j}}$$

$r_{i,j}$ quantifies the relative expression level of gene *i* in the heterogeneous group *j*.

Finally, the activity score $p_{t,j}$ of the *t*-th immune pathway in the *j*-th heterogeneous group is defined as the weighted average of $r_{i,j}$ of all genes contained in the *t*-th immune pathway:

$$p_{t,j}=\frac{\sum_{i=1}^{m_{t}}w_{i}\times r_{i,j}}{\sum_{i=1}^{m_{t}}w_{i}}$$

$m_{t}$ is the number of genes in the *t*-th immune pathway, $w_{i}$ is the weighting factor of the *i*-th gene, and $w_{i}$ is equal to the reciprocal of the number of the *i*-th gene in all immune pathways.

Through the three calculation steps above, we finally obtained the activity score of each immune pathway in three groups.

#### Evaluation of Immune Pathway Score by the Single Sample Gene Set Enrichment Analysis Algorithm

In addition to the method described above, we also used the single sample gene set enrichment analysis (ssGSEA) algorithm to evaluate the activity score of each immune pathway in each sample across different groups (Barbie et al., 2009).

### The Infiltration of Immune Cells in Samples

We obtained 28 immune cells and the related 782 marker genes from Trajanoski Z et al. (Charoentong et al., 2017). These marker genes were expressed in specific immune cells (Supplementary Table S1). Then, the ssGSEA algorithm was used to evaluate the infiltrative level of each immune cell in one sample based on the expression profile of marker genes.

### Identification of Immune Subtypes

Based on the obtained infiltration profile of nine key immune cells, we used the consensus clustering algorithm to identify the immune subtypes of LUAD patients using the “ConsensusClusterPlus” package. The clustering was performed with 1,000 iterations and 80% resampling, and the Pearson correlation coefficient was used as a similar distance. As a result, the samples were found to be clustered into two subtypes with significantly different clinical outcomes.

### Evaluation of the Activity Score for Each Metabolic Pathway

The metabolic pathways and the related genes were also obtained from the KEGG database. Using the ssGSEA algorithm, we calculated the activity score of each metabolic pathway in each tumor sample.

## Results

### The Infiltration of Immune Cells Varies Among Normal, Low-Heterogeneous, and High-Heterogeneous Tumor Samples

**FIGURE 1:**
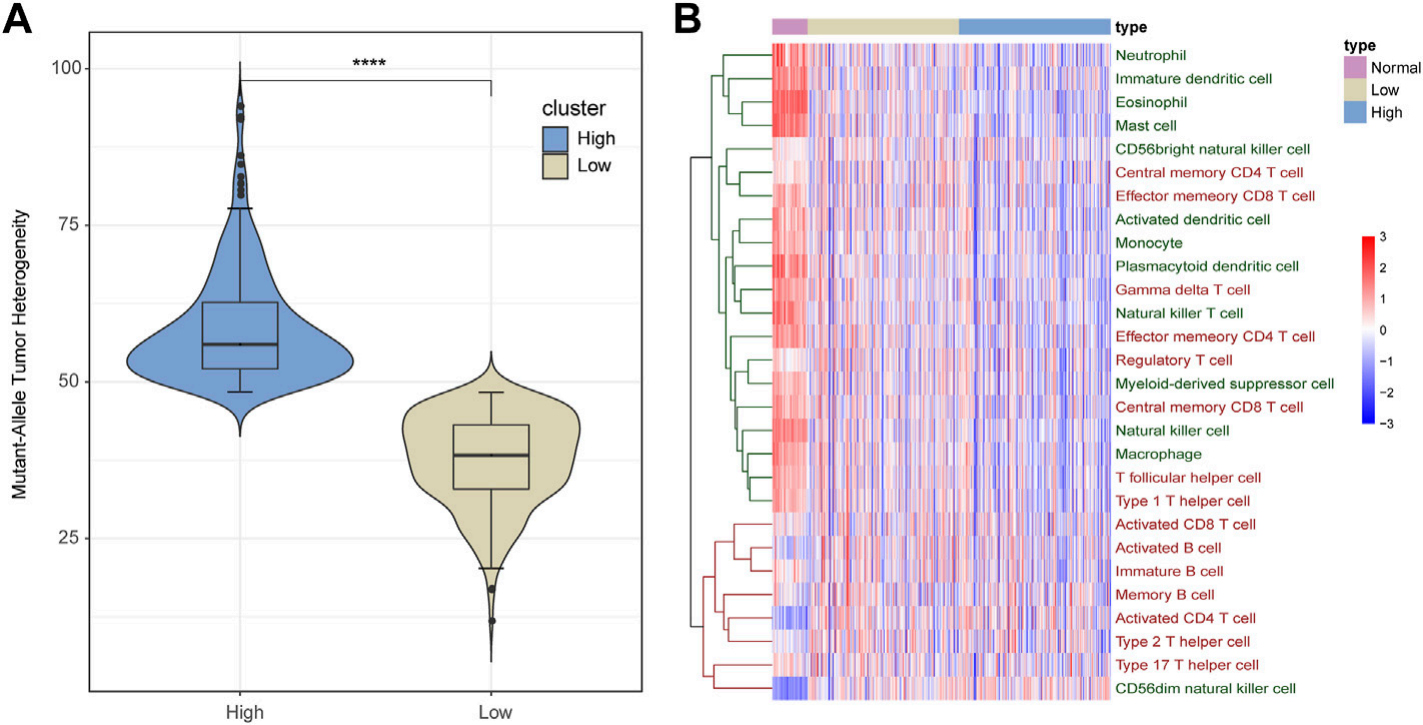
Infiltration of immune cells in LUAD samples. **(A)** All LUAD samples are divided into low- and high-heterogeneous groups based on the MATH score. **(B)** Infiltration of immune cells varies among normal, low-heterogeneous, and high-heterogeneous cancer. Adaptive immune cells are marked red, and innate immune cells are marked green.

Intra-tumoral heterogeneity enables aggressive progression and resistance to treatment and is crucial for proliferation, invasion, and drug resistance of tumor cells. As described in the section “Materials and Methods”, based on the mutation data of LUAD obtained from the TCGA database, we calculated the MATH score of each cancer sample, which represented the heterogeneous degree of tissue samples. According to the median of the MATH score of cancer samples, we divided these samples into low- and high-heterogeneity groups with 254 and 254 samples, respectively (Figure 1A).

Recent studies have found that ITH can affect the ability of the immune system to induce tumor cells to evade immune recognition and killing. Therefore, we calculated the infiltration of 28 immune cells in each normal and cancer sample. As shown in Figure 1B, the infiltration pattern of immune cells could be classified into two groups. Most innate immune cells (12 out of 13) and a portion of adaptive immune cells (8 out of 15, mainly memory T cells) presented a high degree of infiltration in normal samples but medium infiltration in low-heterogeneity tumor samples and a low infiltration level in high-heterogeneity tumor samples. On the other hand, other immune cells including seven adaptive immune cells (B cells, activated T cells, and T helper cells) and one innate immune cell showed low levels of infiltration in normal samples, compared with cancer.

### Five Key Immune Pathways Were Identified to be Most Related to the Cancer Heterogeneity

Immune cells in the TME play a paramount role in tumor development and influence the prognosis of cancer. ITH utilizes various mechanisms to dysregulate immune pathways, thereby affecting the biological function of immune cells (Huang et al., 2020). Therefore, we screened the immune pathways which had different activities among the three groups by two approaches.

**FIGURE 2:**
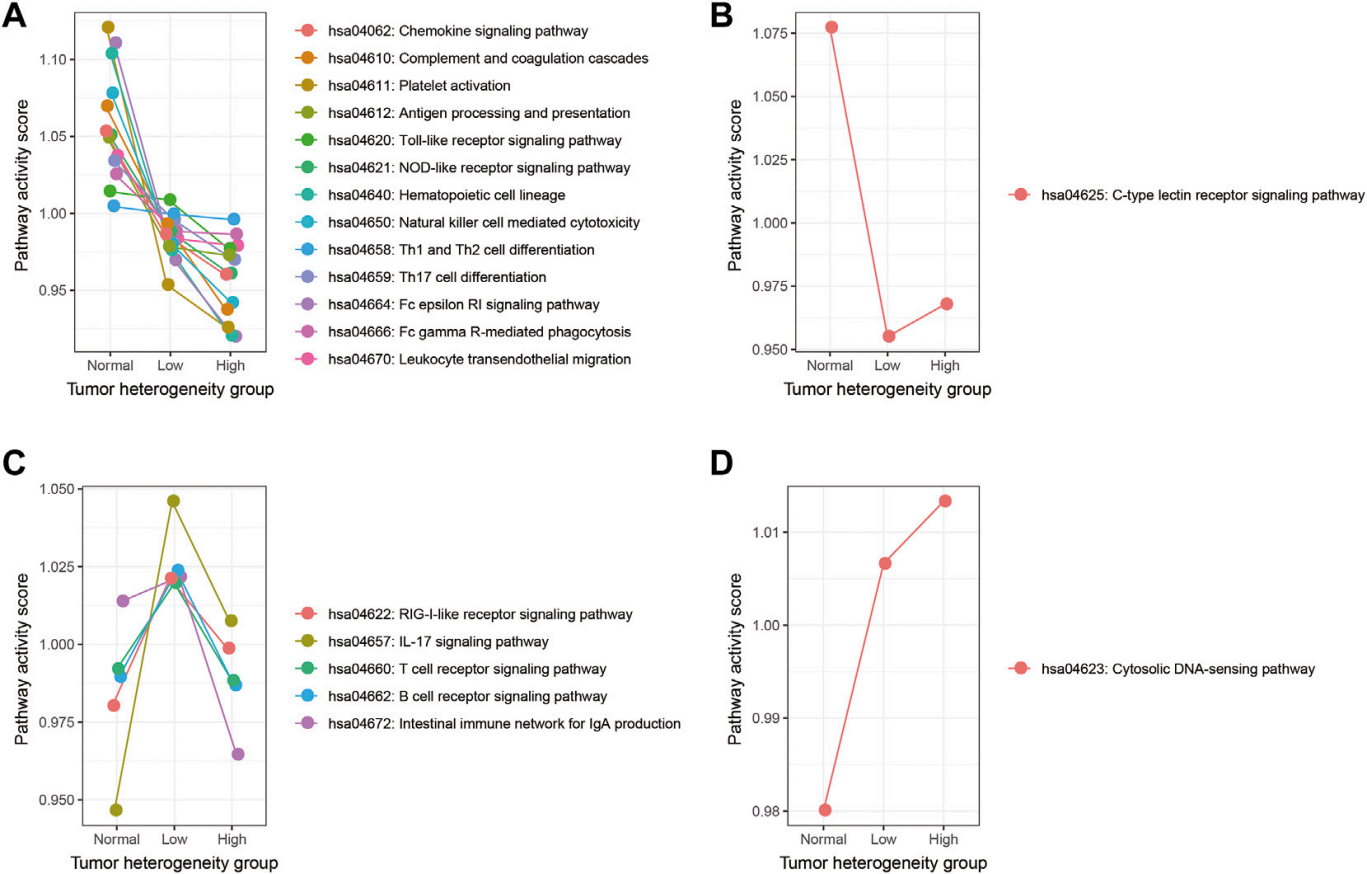
Activity score of each immune pathway in three groups calculated by the Jason W. Locasale method. **(A)** Immune pathways which showed a continuous decreasing trend. **(B)** Immune pathways which showed a downward trend, followed by an upward trend. **(C)** Immune pathways which showed an upward trend, followed by a downward trend. **(D)** Immune pathway which showed a continuous increasing trend.

In the first method, we adjusted the method proposed by Jason W. Locasale et al. to calculate the activity score of each immune pathway in the three groups, as described in the section “Materials and Methods” (Xiao et al., 2019). First, the mean expression of each gene in each immune pathway was calculated in each group. Second, for each gene in a certain immune pathway of each specific group, we calculated the ratio of its expression to the mean expression value of the three groups. The ratio value greater than 1 meant that this gene was activated; otherwise, it is the opposite. Finally, the weighted summation of all genes in one immune pathway was calculated as the activity score of this pathway in a certain group. Comparing the activity scores of immune pathways among the three sample groups, we found that the activity scores of these immune pathways showed four types of variation trends, with 13 out of 20 immune pathways presenting a continuous decrease in normal, low-heterogeneous, and high-heterogeneous samples (Figure 2).

**FIGURE 3:**
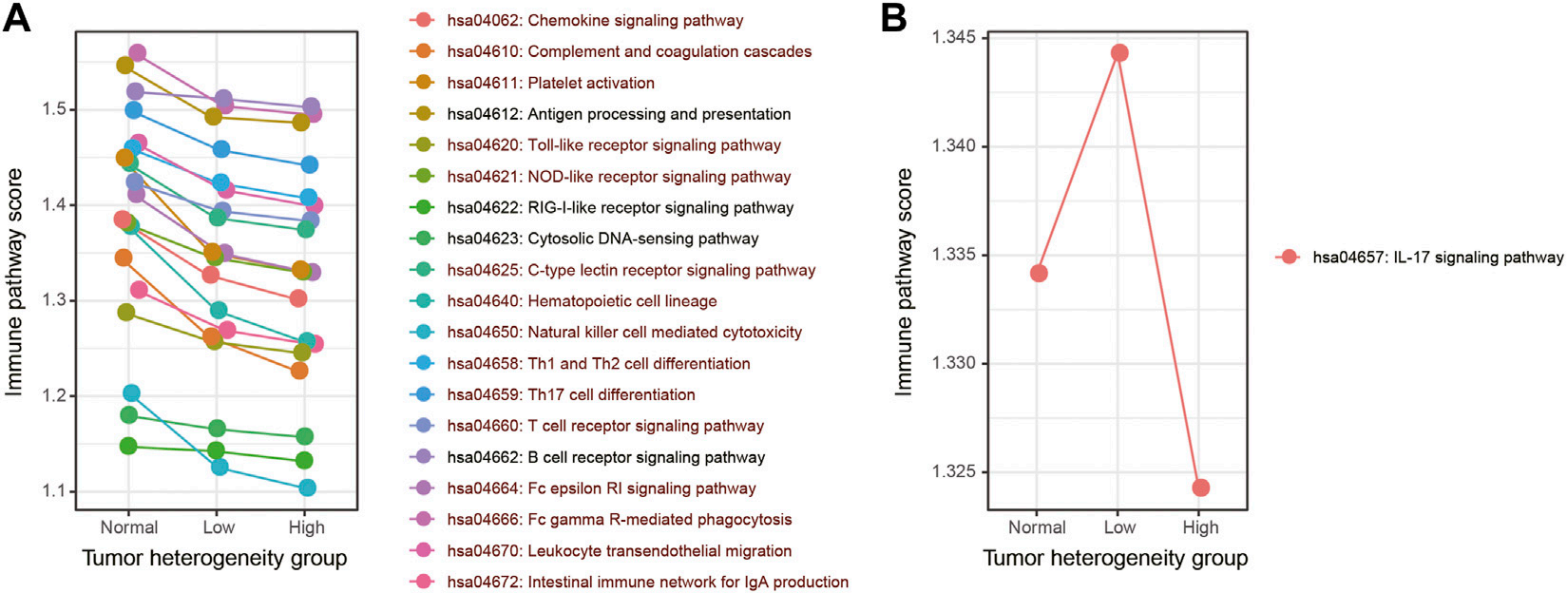
Activity score of each immune pathway in three groups calculated by the ssGSEA algorithm. **(A)** Immune pathways which showed a continuous decreasing trend. **(B)** Immune pathways which showed an upward trend, followed by a downward trend. The immune pathways whose activity scores have significant differences among three groups are marked in dark red.

In the second method, we used the ssGSEA algorithm to obtain the activity score of each immune pathway in the three groups (Figure 3). As a result, 19 out of 20 immune pathways showed a continuous decreasing trend in normal, low-heterogeneous, and high-heterogeneous sample tissues (Figure 3A). However, only the IL-17 signaling pathway showed an upward trend, followed by a downward trend (Figure 3B). Then, the Wilcoxon rank sum test was used to compare the ssGSEA scores of immune pathways among the groups. A total of 15 immune pathways were identified with a significant difference among the three groups, of which the activity scores were all continuously decreased in normal, low-heterogeneous, and high-heterogeneous samples.

**FIGURE 4:**
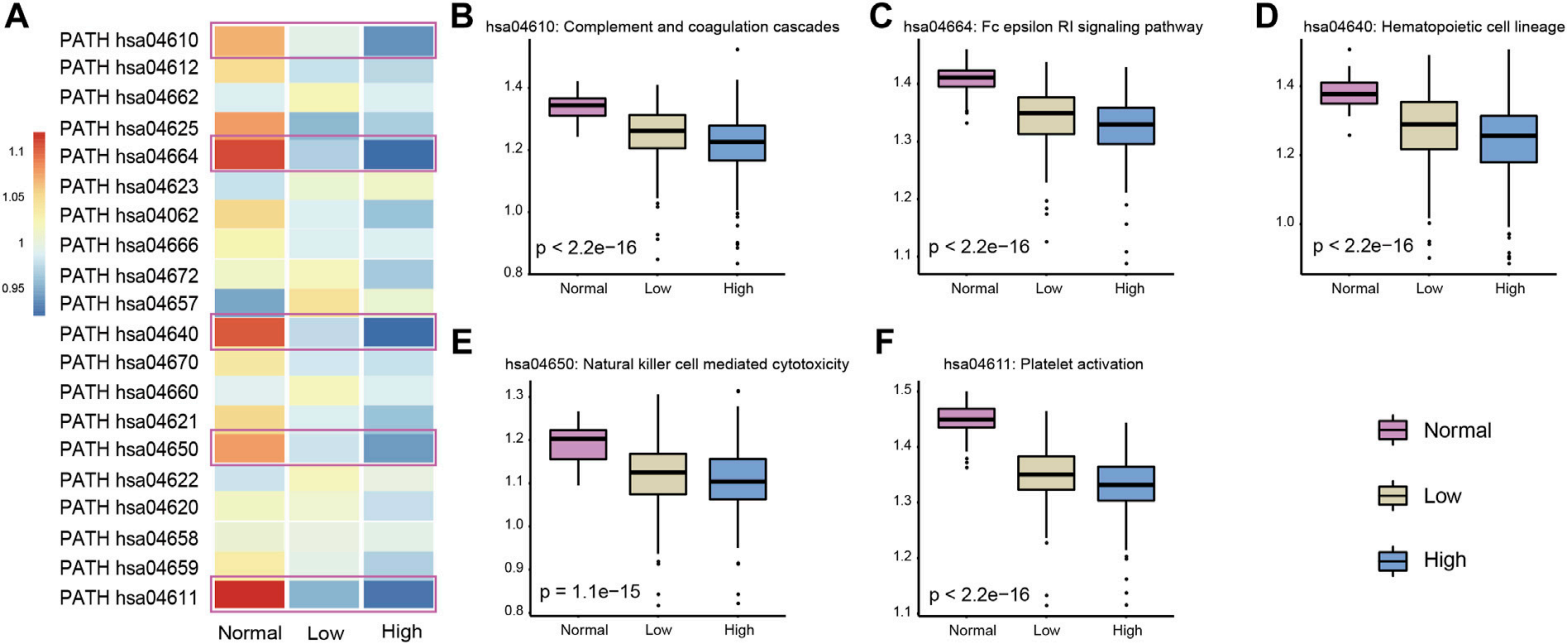
Selected five immune pathways were most related to the LUAD cancer status. **(A)** Activity score of each immune pathway in three groups was calculated by the Jason W. Locasale method. The selected five immune pathways were marked with boxes. **(B–F)** Activity score of five immune pathways in three groups was calculated by the ssGSEA algorithm. *p*-values were obtained by Kruskal–Wallis.

Integration of these two approaches revealed that those immune pathways which presented a continuous decreasing trend from normal samples to low- and high-heterogeneous cancer samples might play important roles in carcinogenesis and cancer development. We selected five key immune pathways for subsequence analysis. These five immune pathways showed the greatest changes in immune pathway activity scores evaluated by the first method (Figure 4A) and presented a significant difference of the ssGSEA scores among the three sample groups obtained by the second method (Figures 4B–F). The five selected immune pathways included complement and coagulation cascades (hsa04610), platelet activation (hsa04611), hematopoietic cell lineage (hsa04640), natural killer cell mediated cytotoxicity (hsa04650), and the Fc epsilon RI signaling pathway (hsa04664). Apparently, all these five immune pathways showed the continuous decreased activity scores. These results were consistent with previous studies (Wu et al., 2020). In addition, tumor samples with high heterogeneity usually showed attenuated immunological competence and have poor clinical outcomes.

Complement and coagulation cascades is a mediator of innate immunity in plasma and plays an important role in wound repair and hemostasis. Platelet activation mediates inflammatory and immunomodulatory activity and plays an essential role in intercellular interactions. Hematopoietic cells can self-renew or differentiate into multi-lineage stereotype progenitor cells. Hematopoietic cells have been found to be involved in the occurrence and resolution of inflammatory events. It is well known that inflammation and cancer are inextricably linked; we believe that this pathway plays vital roles in cancer. Natural killer cells are lymphocytes of the innate immune system and can target and kill abnormal cells, such as virus-infected and tumorigenic cells. In tumor samples with higher heterogeneity, their activity was found to be decreased in our study. The FcεRI compound can control the secretion of allergenic mediators and induce the transcription of cytokine genes by inducing and activating a variety of signaling pathways and further influence the inflammatory response and carcinogenesis. In summary, the five immune pathways identified in our study were significantly associated with ITH.

### Identification of the Nine Key Immune Cells Related to the Key Immune Pathways and Tumor Heterogeneity

**FIGURE 5:**
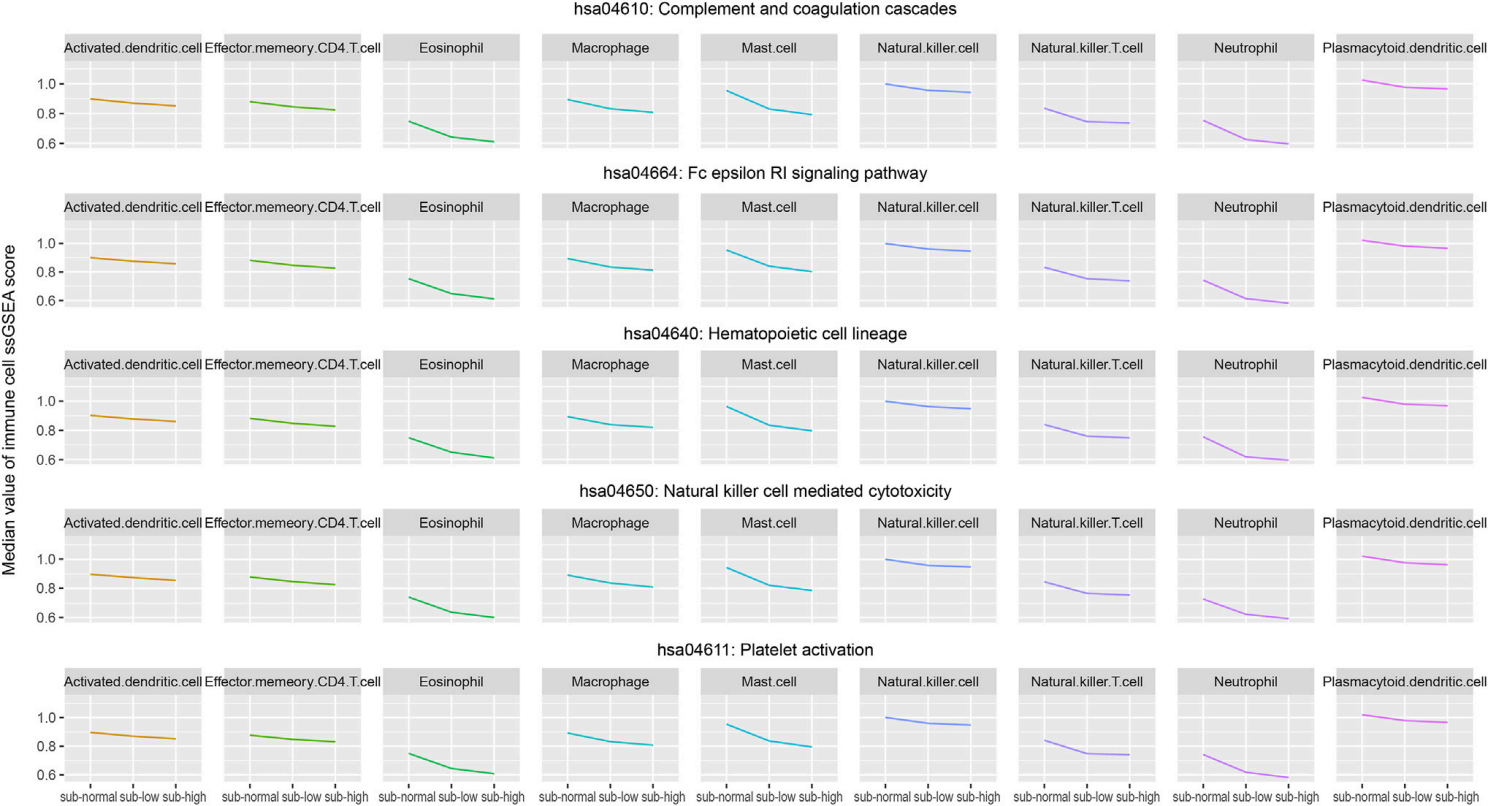
Median values of infiltration score of the selected nine immune cells in the three groups (subnormal, sub-low-heterogeneous, and sub-high-heterogenous tumor samples) were evaluated by the ssGSEA algorithm.

Based on the expression profile of 782 marker genes of 28 immune cells, using the ssGSEA algorithm, we calculated the infiltration score of each immune cell in each normal and cancer sample. For the three sample groups (normal, low-heterogeneous, and high-heterogeneous groups), we further selected those samples with higher ssGSEA activity scores of key immune pathways as the new subgroups, including subnormal, sub-low-heterogeneous, and sub-high-heterogeneous samples. For each key immune pathway, the samples in the new subgroups have relatively higher activity scores. We analyzed the dynamic change of immune cell scores in subgroups with higher pathway scores among the three sample groups, which could identify more effective immune cells. Then, the infiltration of immune cells was evaluated among the new subgroups. Those cells with significantly different infiltration among the subgroups were selected as the key immune pathway-related immune cells (Wilcoxon test *p* < 0.05 between each two subgroups, Kruskal–Wallis test *p* < 0.05 among the three subgroups). Furthermore, we evaluated the correlation of immune cell infiltration with sample heterogeneity using the Pearson correlation test. Those cells with R < −0.4 and BH-FDR < 0.01 were selected as the heterogeneity-related immune cells. Integrating the results of key immune pathways and heterogeneity-related immune cells, we finally identified nine key immune cells, including activated dendritic cells, effector memory CD4 T cells, eosinophils, macrophages, mast cells, natural killer cells, natural killer T cells, neutrophils, and plasmacytoid dendritic cells (Figure 5). We suppose that these nine key immune cells may have essential roles in the cause and development of lung adenocarcinoma.

Notably, eight out of nine key immune cells are innate immune cells. Only the effector memory CD4 T cell is the adaptive immune cell. The innate immune system is a natural immune defense mechanism in the human body and is essential for human immunity. Innate immune cells also play an indispensable role in immunotherapy. For example, dendritic cells can present tumor antigens to CD8 T cells, activate specific CD8 T cells, and kill tumor cells. Most innate immune cells have a profound influence on the immune pathway. Generally, the nine key immune cells could be divided into three classes, including antigen presentation cells, cells interacting with effector T cells, and cells functioning similar to effector T cells. Antigen presentation cells, such as activated dendritic cells, eosinophils, and plasmacytoid dendritic cells, can present the identified antigen to effector cells to contribute to the identification and function of the effector cells. The second class of cells, such as macrophages, mast cells, and neutrophils, usually plays roles with effector T cells. Other cells, such as effector memory CD4 T cells, natural killer cells, and natural killer T cells, have the ability to kill cells and perform similar functions with effector T cells.

### The Immune Subtypes Identified Based on the Key Immune Cells

**FIGURE 6:**
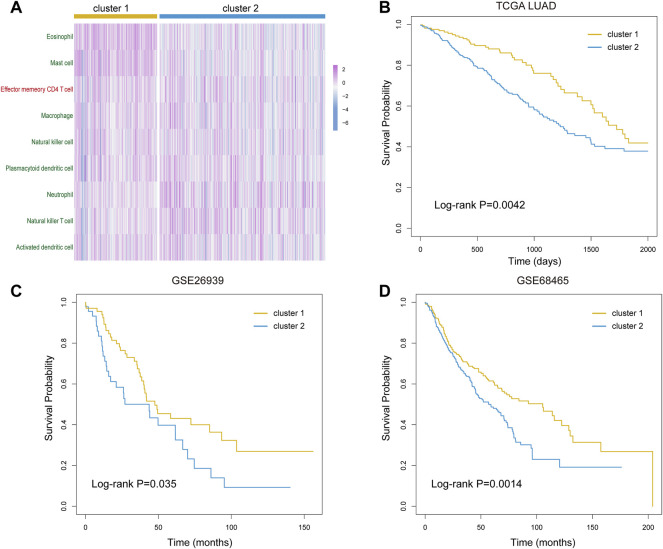
Prognostic evaluation of the selected nine immune cells. **(A)** Infiltration profile of the selected nine immune cells in cluster 1 and cluster 2 groups. **(B–D)** Kaplan–Meier curves of overall survival in cluster 1 and cluster 2 groups of LUAD patients from TCGA **(B)**, GSE26939 dataset **(C)**, and GSE68465 dataset **(D)**.

As the key immune cells were related to the ITH and key immune pathways, we hold the opinion that the infiltrative level of them is vital in cancer development. Next, we used the consensus clustering algorithm to identify the immune subtypes for LUAD patients based on the infiltration profile of the nine key immune cells. According to the clustering result, the samples were divided into two subtypes. Key immune cells showed differential infiltration in these two sample groups (Figure 6A). Furthermore, the survival analysis showed that the clinical outcome of the two groups had significant differences (Log-rank *p* = 0.0042, Figure 6B). This result suggested that our identified nine key immune cells play essential roles in cancer progress and contribute to the prognosis of lung adenocarcinoma.

To verify our results, we next downloaded and analyzed two GEO datasets of lung adenocarcinoma. After data pretreatment of expression profiles, we calculated the infiltration of nine key immune cells in tumor samples using the ssGSEA algorithm in two GEO datasets. We obtained two similar subtypes by the consensus clustering algorithm and compared the overall survival probability between subtypes for the two datasets. As a result, the clinical outcome of two subtypes presented significant differences (Log-rank *p* = 0.035 for GSE26939 and 0.0014 for GSE68465, Figures 6C,D). These results confirmed the importance of our identified key immune cells and the robustness of our results.

### The Genomic Difference Between Two Immune Subtypes

**FIGURE 7:**
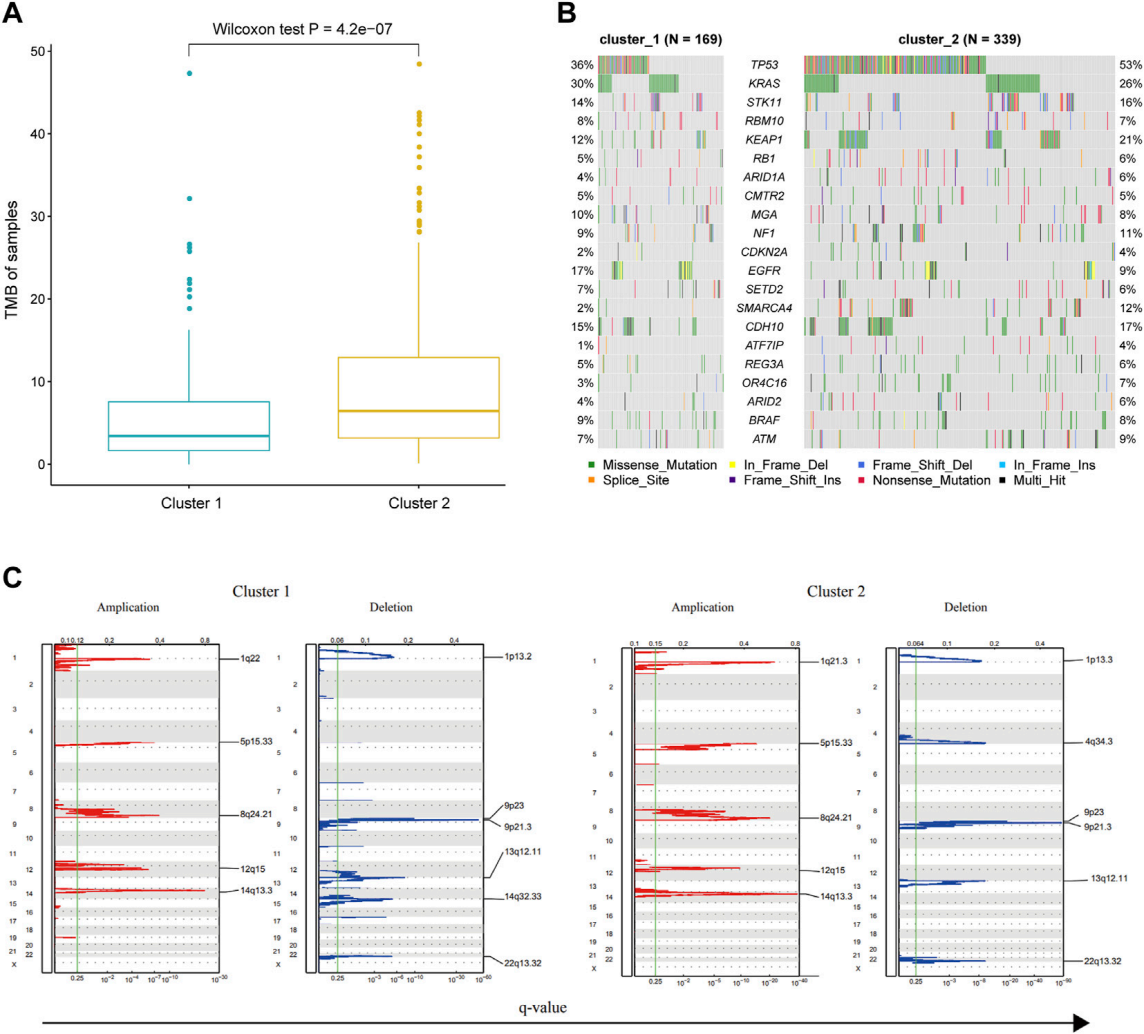
Genomic difference between the samples of cluster 1 and cluster 2 groups. **(A)** TMB of tumor samples in each immune subtype. **(B)** Comparison of significant mutated genes in different immune subtypes. **(C)** Comparison of CNV, including genome implication and deletion, between two immune subtypes.

TMB distribution of immune cells as predictive biomarkers plays an important role in evaluating the efficacy of targeted therapy and immunotherapy. Some studies have shown that the infiltrating macrophage was related to the immunophenotype, TMB, and clinical prognosis of LUAD patients (Ma et al., 2021). Furthermore, the combination of macrophages and TMB could further improve the prediction accuracy. Therefore, we compared the distribution of TMB for LUAD samples in two different immune subtypes. The somatic mutation data of LUAD were used to calculate the TMB for each sample. We then compared the differences in TMB between the two immune subtypes using the Wilcoxon rank-sum test. The results showed that the TMB level of the cluster 2 subtype was significantly higher than that of the cluster 1 subtype (Wilcoxon test *p* = 4.2e-07, Figure 7A). Recent studies have shown that patients with high TMB were more likely to benefit from immunotherapy (Rizvi et al., 2015). This implicated that the cluster 2 subtype might have a better clinical response to immunotherapy. In this study, by adopting the dNdScv method, we identified 21 significantly mutated genes (SMGs) in LUAD (Figure 7B). Among these genes, *TP53*, *KEAP1*, and *SMARCA4* had a high mutation frequency in the cluster 2 subtype. At the same time, a high mutation frequency of the *EGFR* gene was observed in the cluster 1 subtype, indicating that tumors bearing cluster 1 subtype immune cells might be more sensitive to EGFR-targeted therapy. Thus, determining the proportion of gene mutation burden of cancer patients may provide guidance for the clinical treatment of patients.

CNV frequently occurs during cancer development and may affect patient response to immunotherapy. Therefore, we classified the CNV data from the TCGA database according to the group information of immune subtypes. Subsequently, we evaluated the CNV for each immune subtype using GISTIC software. The result showed that the significantly amplified or deleted regions were generally consistent in both immune subtypes. However, there were several exceptions, such as the amplification in 1q22 in the cluster 1 group, the amplification in 1q21.3 in the cluster 2 group, the deletion in 1p13.2 and 14q32.33 in the cluster 1 group, and the deletion in 1p13.3 and 4q34.3 in the cluster 2 group (Figure 7C). Previous studies have shown that expression levels of BCL10 and GFI1 in the 1q22 region of the cluster 1 subtype could affect the biological function of T cells (Rosenbaum et al., 2019), and the expression levels of F3 in the 1q21.3 region of the cluster 2 subtype could strengthen the cytotoxicity of killer cells (Chu et al., 1990). These data suggested that CNV may account for the different clinical outcomes of the two immune subtypes.

### The Different Roles of Metabolic Pathways in the Two Immune Subtypes

**FIGURE 8:**
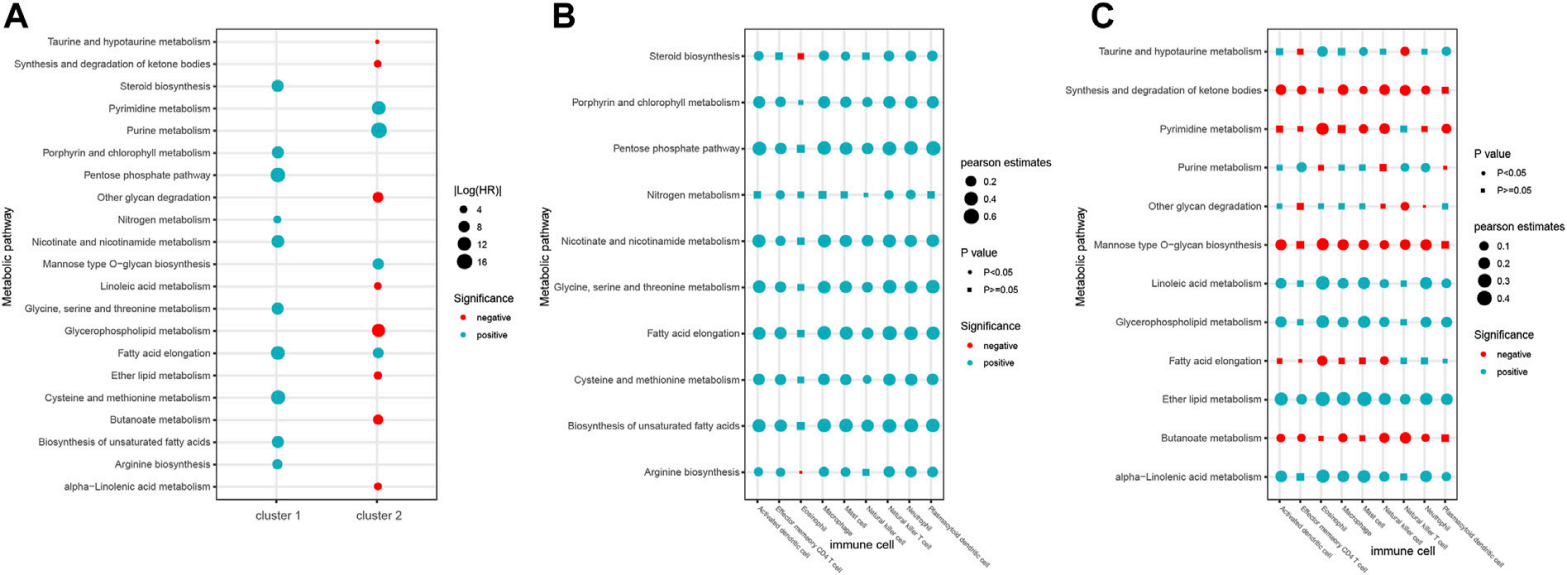
Roles of metabolic pathways in two immune subtypes. **(A)** Prognosis of metabolic pathways in two immune subtypes. **(B–C)** Correlation between metabolic pathways and nine immune cells of immune cluster 1 **(B)** and cluster 2 **(C)**.

Substantial evidence has shown that most tumors emerge and evolve under selective pressure involving metabolic and immune reconstruction (Vitale et al., 2019; Jiang et al., 2022). For example, highly glycolytic cancer cells can redirect glucose to anabolic responses for supporting proliferation and also exert immunosuppressive effects by secreting more lactate (Zhu and Thompson, 2019). Therefore, we calculated the activity scores of each metabolic pathway in all samples and evaluated its contribution to the clinical prognosis by the univariate Cox regression analysis. In total, we identified 21 metabolic pathways that were significantly associated with clinical outcomes of patients (cox *p* < 0.05, Figure 8A). Among them, 10 metabolic pathways were found to be associated with clinical outcomes in the cluster 1 subtype. Except for fatty acid elongation, other metabolic pathways were unique to the cluster 1 subtype (Figure 8A). In the cluster 2 subtype, there were a total of 12 metabolic pathways related to patient outcomes, and 11 metabolic pathways were unique to cluster 2. Furthermore, we found that all 10 metabolic pathways in the cluster 1 subtype were risk factors (HR > 1), while most of the pathways in the cluster 2 subtype were mainly protective factors (HR < 1). These results suggest that different metabolic pathways may help to determine the characteristics of two immune subtypes.

Recent studies have shown that dysregulation of metabolic pathways can affect the behavior of immune cells, leading to immune dysfunction. Then, we analyzed the association between the activity of metabolic pathways and the infiltration of nine immune cell types in the two different immune subtypes. Consequently, we found that eight immune cells except the eosinophil showed significant positive correlation with most of the 10 metabolic pathways in the cluster 1 subtype (Figure 8B, *p* < 0.05). In the cluster 2 subtype, five metabolic pathways including synthesis and degradation of ketone bodies, mannose type O-glycan biosynthesis, pyrimidine metabolism, fatty acid elongation, and butanoate metabolism were negatively correlated with immune cells, while other seven metabolic pathways were positively correlated with immune cells (Figure 8C, *p* < 0.05). These results suggest that the correlation between individual immune cells and metabolic pathways in the two immune subtypes is helpful to better understand the complexity of the immune metabolism.

## Discussion

ITH plays an important role in tumor cell growth, metastasis, resistance to chemotherapy, and immunotherapy of LUNG. The diversity of tumor cells in the TME contributes to ITH (Prasetyanti and Medema, 2017). However, few studies have investigated the crosstalk between tumor cells and immune cells within the TME. In this study, we identified five key immune-related pathways in different heterogeneous groups of LUAD samples and further found nine differential immune cells related to the above immune pathways. Subsequently, we further characterized two immune subtypes, of which different genomic features such as TMB and metabolic pathways interacted with immune cells functionally.

Immune pathways have been found to be an intermediate between tumor cells and immune cells (DeCordova et al., 2020). In order to obtain more robust results, we currently adopted two computational methods of pathway score, the Jason W. Locasale method and ssGSEA algorithm, but not the commonly used CIBERSORT method. We identified nine types of immune cells that differed significantly in three heterogeneous subtypes and were also significantly associated with the ITH status in LUAD. Most of these immune cells belong to nonspecific immune cells, which is essential for human immunity. Notably, some pieces of evidence have suggested that immunotherapy approaches have significant effects on both spatial and temporal ITH (Gubin et al., 2018). Meanwhile, the clinical efficacy of immune checkpoint inhibitors may involve multiple immune cell populations in addition to cytotoxic T lymphocytes. Therefore, it is necessary to elucidate the exact influence of immune cell heterogenization on tumor heterogeneity. In our results, a total of nine immune cells were identified, which were mainly divided into three main categories, including antigen-presenting cells, cells that interact with effector T cells, and cells with similar functions to effector T cells. The score of these immune cells decreased with the increase of tumor heterogeneity.

Some studies have found that ITH is associated with the clinical features and prognosis of LUAD samples. Nonetheless, the ITH was not found to be different across four disease stages (Wilcox *p* > 0.05) in our study. This suggests that ITH may not increase as cancer cell progresses. However, from a genetic perspective, the accumulation of genomic mutations drives cancer initiation. Additionally, several studies have found significant differences in the TMB level and CNVs in non-small-cell carcinoma tumor samples with different ITH (Zhao et al., 2019; Craig et al., 2022). Similarly, our analysis also showed that TMB and MATH scores were higher in the cluster 2 subtype than in the cluster 1 subtype. Furthermore, most of the regions that were significantly amplified or deleted were generally consistent in both immune subtypes, with a few exceptions. The crosstalk between ITH and immune cells plays an important role in shaping the biological phenotypes of tumor cells, resulting in endogenous inconsistency in immunotherapy responses.

In summary, we identified two new immune subtypes by integrative evaluation of the ITH, immune-related pathways, and infiltrating immune cell status of LUAD patients. We found that these immune subtypes were characterized by differences in mutation burden, CNV, and clinical outcomes of patients. Taken together, the present study has provided a further understanding of the complex relationships between tumor cells and infiltrating immune cells in LUAD and will contribute to the development of personalized therapy.

## Data Availability

The original contributions presented in the study are included in the article/Supplementary Material; further inquiries can be directed to the corresponding authors.
